# Newspaper Reading in Families with School-Age Children: Relationship between Parent–Child Interaction Using Newspaper, Reading Motivation, and Academic Achievement

**DOI:** 10.3390/ijerph192114423

**Published:** 2022-11-03

**Authors:** Naya Choi, Jiyeon Sheo, Suji Jung, Jisu Choi

**Affiliations:** 1 Department of Child Development and Family Studies, Seoul National University, Seoul 03080, Korea; 2 Department of Psychology, University of California, San Diego, CA 92039, USA

**Keywords:** newspaper subscription, newspaper reading, parent–child interaction, reading motivation, academic achievement

## Abstract

The present study aims to observe the patterns of newspaper subscription and reading and further explore the structural relationship between parent–child interactions, children’s reading motivation, and academic achievement in families with school-age children. Online surveys were administered to 1361 parents of elementary students from grade 1 to 6 across South Korea. Collected data were analyzed using SPSS and AMOS to conduct frequency analysis, correlation analysis, structural equation modeling, and bootstrapping analysis. Results showed the following. First, 17.0% of households subscribed to a newspaper, 28.5% of parents read paper newspapers, and 97.1% of parents read online newspapers. Second, parent–child interaction using newspapers had an indirect effect on children’s academic achievement through the mediating effect of reading motivation. Overall results revealed the functions of newspaper as part of home literacy environment and the newspaper’s positive contribution to a child’s reading motivation and academic achievement.

## 1. Introduction

As one of the most easily accessible literacy resources in homes, newspapers engage young readers with a diverse array of information about the world. Various studies have revealed positive effects of newspapers on children’s literacy development and highlighted the importance of newspaper subscriptions, presentation, and reading as crucial variables [1,2,3]. Studies that have tested the educational effects of newspapers have also shown that they promote students’ academic interests and motivations [4], along with academic achievement in various subjects [2,4,5,6,7,8,9]. However, since the emergence of digital media, there has been a shift in news consumption patterns. Hence, this work attempts to explore the current patterns in newspaper subscription and reading and further broaden the focus by examining the relations between parent–child interactions using newspapers, the child’s reading motivation, and academic achievement.

Newspapers as distinct learning tools have been integrated in classrooms around the globe since the 1970s under the name of “Newspaper in Education, NIE” [10]. As one of the earliest recognized benefits of newspapers being their application in reading, newspapers quickly became part of the school curriculum. Beginning with the United States, major newspaper companies in Canada and Europe also took part in the NIE program by distributing newspapers as educational material free of charge [11]. Newspapers were further used to facilitate students’ literacy, critical thinking, and address specific classroom learning themes [12].

Compared to other literacy devices, newspapers contain real-world informational content creating diverse learning contexts. Newspapers also have distinct physical characteristics that allow for easier handling by young children. The positive implications of newspapers have been explored until relatively recently in the education realm, including academic subjects on language arts [2,4], science [5,13], social studies [6], English [7], and math [8,9]. Studies continue to show how newspapers as learning tools can provide additional support for school children. While past literature makes a strong case for the positive impact of newspapers in classrooms, there is a paucity of research revealing the role of newspapers outside such contexts. Considering that elementary school children usually become acquainted with literacy at home [1,14], more research is needed to explore the role of newspapers in home environments.

## 2. Background Literature

### 2.1. Current Trend in Newspaper Reading

The demand for print newspapers has declined naturally over time. Rapid digitalization has led to drastic changes in newspaper reading patterns [15]. While public interest in print newspapers and newspaper subscription rates have declined significantly, online newspapers have received widespread attention. A report published by the Pew Research Center showed that the number of weekly publications in the United States dropped over half from 55,773,000 in 2000 to 28,554,137 in 2018. In Japan, the annual circulation of paper newspapers decreased by more than 10 million copies from 2000 to 2018 [16]. A similar phenomenon was evident in South Korea. According to a public survey conducted in 2017 [17], the paper newspaper subscription rate was 19.8%, and the readership rate was 33.5%, indicating the low utilization of paper newspapers. In contrast, the online newspaper usage rate was 73.2%. While there was an overall drop of interest in paper newspapers, the opposite was found for online newspapers. In light of such changes in media consumption patterns, there is a need to examine the current status of newspaper subscriptions and readership.

Furthermore, only a few existing studies have examined children’s use of newspapers and information on parent–child interactions utilizing newspapers is even more scarce. A survey in 2015 by the National Literacy Trust investigated literacy behaviors of children from ages 8 to 16. Of the participants, 26.2% responded that they read paper newspapers outside classrooms and 34.1% read newspapers on tablets [18], indicating that children engage in leisure newspaper reading outside the classroom in environments such as homes. However, the research is relatively old and the surrounding context in which newspapers are read remains unclear. Engaging with reading outside the school setting has been found to promote literacy, reading competence, and motivation [19]. To implement newspapers as intervention measures in children’s learning environments, updated information on the use of newspapers by families with elementary school students as early readers of newspapers is warranted.

### 2.2. Parent–Child Interaction Using Newspaper, Reading Motivation, and Academic Achievement

Past literature has consistently shown that home literacy environments are crucial for children’s language and cognitive development [1,14,20,21,22]. The home literacy environment (HLE) is a broad term that refers to not only physical components, but also literacy-related parent–child interactions [23,24,25]. As elementary school students are influenced by various factors in the learning process, HLE plays a significant role in helping school-aged children, who are undergoing rapid cognitive developmental changes.

According to Vygotsky [26], children’s development takes place in a sociocultural context in which social interactions with primary others, such as parents, have a profound impact. Wasik and Herrmann [27] categorized four variables related to children’s literacy development and noted parent–child interactions as the most dominant variable. Yoon [28] also reported in his study among Korean students that home environmental factors showed the strongest effect on children’s educational achievement. Extending the social interactional framework, parent–child interactions using newspapers as part of the HLE can be predicted to have a positive impact on children’s development. Yet, only a few studies have used this framework to examine literacy interactions using newspapers [29,30].

In Yoo and Jung’s study [29], the authors categorized parental mediation types during newspaper reading into “restrictive/instructional mediation” and “explanatory/discussive mediation.” In the first type, parents gave instructions on what to read and what not to read, with an assigned reading time. The latter type involves a more active learning process in which the child and parent engage in a discussion together after co-reading the newspaper. Parental guidance was only provided to clarify the child’s understanding. In another study, Choi and Jeong [30] developed the Home Newspaper Utilization Scale for elementary school students with specific types of parent–child interactions including online mediation, conversation, play activity, and scrap activity using newspapers. This scale is one of the few scales that include online interaction, which is particularly important in light of the recent newspaper trend. Although both studies focus on investigating the diverse parental behaviors during parent–child interaction using newspapers, neither study shows how parent–child interactions via newspapers are associated with children’s cognitive development. Therefore, this study addresses the use of newspapers at home and its relationship with academic performance.

Furthermore, elementary school children’s academic achievement as a developmental index for examining language and cognitive development has consistently attracted researchers’ attention. Additionally, academic achievement is now used as a predictor of school adaptation, sociability, and a child’s overall quality of life [31]. Academic achievement has also been found to affect a child’s overall life satisfaction and to be associated with healthy development throughout adolescence and adulthood [32,33]. Hence, it is vital to assess variables that affect academic performance and maximize educational effectiveness.

Studies have further highlighted associations between the HLE and children’s linguistic and cognitive domains. Parental modeling behavior using literacy material, such as newspapers, was found to be related with their children’s verbal abilities [1,20]. In addition, children who received higher parental verbal support during the parent–child interaction showed higher reading-related skills [21,25]. According to a study on first-year elementary school students, HLE was shown to have a positive correlation with language performance [22]. In another three-year longitudinal study among five-year-old children, a higher quality HLE was associated with higher academic achievement [1]. Although the above studies reveal the relationship between HLE and children’s academic performance, the underlying mechanisms remain unclear. Hence, there is a need to understand through what pathways interactions lead to improved academic achievement.

HLE has also been closely associated with children’s reading motivation, a psychological factor that sustains student’s reading activities and regulates reading-related behaviors [34]. Reading skills are an important developmental task for elementary school students who need to have a wide range of new literacy experiences. In this case, reading motivation acts as a facilitator to maintain reading habits. In a study on Chinese children, the higher the number and volume of reading instances modelled by parents, the higher the child’s reading motivation [35]. In a study on American children, the higher the number of reading interactions at home, the higher the child’s reading motivation [36]. In addition, the HLE for five-year-old children in South Korea mediated by reading experience affected both reading motivation and reading abilities [34]. As children exposed to a rich HLE are reported to have higher reading motivation than children who are not, parental interaction using newspapers may also be an important part of the literacy environment.

Researchers have also consistently shown that reading motivation is closely related to academic performance. Middle school students who spend at least six hours a week reading books showed higher academic performance [37], and fifth graders’ voluntary reading outside of school had a 16% higher impact on reading skills compared to kids who did not engage in voluntary reading. It was also reported that students with strong reading motivation show higher academic performance [38] and use more diverse cognitive strategies [39]. Taken together, studies suggest reading motivation as a possible variable in explaining the linkage between parent–child interaction and academic achievement.

Existing studies have not yet examined the association between parent–child interaction using newspapers and academic achievement while considering reading motivation as an underlying variable. Past studies have been predominantly limited to verifying newspaper effects on particular school subjects, and to our knowledge, no recent research has been conducted to update the current newspaper subscription rates and reading patterns in families with young children. Therefore, in this study, we attempt to broaden our understanding on the role of newspapers in families with school-aged children. In addition, based on the previous discussions, we verify the mediating effect of children’s reading motivation between parent–child interactions and academic performance by using a structural equation model. In the midst of a widening array of media services available, this study has important implications for digital-age children. Through our study, we wish to provide practical guidelines to utilize newspapers as an alternative educational material and lay the foundations for developing home NIE programs to foster a positive learning environment.

The research questions proposed are as follows.

1. What is the status of newspaper subscriptions and reading at home with school-age children?
2. What is the correlation between parent–child interactions using newspapers, children’s reading motivations, and academic achievements?
3. What is the structural relationship between parent–child interactions using newspapers, children’s reading motivations, and academic achievements?

## 3. Materials and Methods

### 3.1. Subjects

Online questionnaires were administered to parents of elementary school children in Seoul, Gyeonggi, Incheon, Gyeongsang, Chungcheong, Jeolla, Gangwon, and Jeju regions across South Korea. A total of 1361 responses were collected; the analyzed number of data differed accordingly to the research questions. First, 1361 household data was used to explore the current status of newspaper subscription and readership (research question 1). Second, of the 1361 households, 238 households with children of third to sixth grade subscribed to either adult or children newspaper were used to examine the correlation between parent–child interactions using newspapers, children’s reading motivation, and academic achievement. Households subscribed to print newspaper were selected as participants as majority of interactional activities require the physicial presence of newspapers. First and second graders were excluded from the analysis as social studies, science, and English (subfactors of academic achievement) are not part of the first and second graders’ curriculum (research question 2). Lastly, the same participants from research question 2 were analyzed to identify the structural relationship between parent–child interactions using newspapers, children’s reading motivation, and academic achievement (research question 3). Table 1 shows the sociodemographic characteristics of 1361 participants.

### 3.2. Data Collection

Participants were selected by using the convenience sampling method because online questionnaires were administered without a list. Convenience sampling is the most common non-probability sampling method adopted to collect research data from the available pool of respondents. This sampling method is efficient and economical in gathering a large number of participants [40]. Researchers distributed survey links on various online communities and social media channels. Subjects voluntarily participated in the study through the distributed links. All participants submitted online informed consent forms.

### 3.3. Measures

Parents’ self-reported questionnaires were used to assess the household’s newspaper subscription and readership, parent–child interaction using newspaper, children’s reading motivation, academic achievement, and sociodemographic information.

#### 3.3.1. Newspaper Subscription and Reading

The household’s newspaper subscription and readership status were measured with separate statements each for the parent and the child. A total of six statements were devised (i.e., “*Are you currently subscribed to print newspaper*?”, “*Are you currently subscribed to children’s newspaper*?”, “*Do you/Does your child read newspaper*?”, “*Do you/Does your child read online newspaper*?”). All six statements were coded as 1 for yes and 2 for no.

#### 3.3.2. Parent–Child Interaction Using Newspaper

Parent–child interaction was measured with the Home Newspaper Utilization Scale for Elementary School Students (HNUS-E) developed by Choi and Jeong [30]. The HNUS-E categorizes interactional behaviors with 3 factors and 9 subfactors. Of the three factors, only one factor (‘Activities’) which focuses on behavior-based interactional activities was used for this study. The ‘Activities’ factor consists of 18 items and 4 subfactors: play activity (i.e., “*I play games using newspapers with my child*”), conversation (i.e., “*I discuss newspaper articles with my child after reading together*”), online mediation (i.e., “*I teach my child how to browse and read online newspaper*”), and scrap activity (i.e., “*I read and scrap newspaper articles with my child*”). All items were evaluated on a 5-point Likert scale (1: strongly disagree, 5: strongly agree). A higher score means a higher parent–child interactional level. Cronbach’s α coefficients of the subfactors are as follows: play activity (0.96), conversation (0.97), online mediation (0.96), and scrap activity (0.94).

#### 3.3.3. Reading Motivation

Children’s reading motivation was assessed with the Reading Motivation Scale for Elementary School Students devised by Jeong and Choi [41]. The scale consists of 3 subfactors: reading attitude (i.e., “*I can fully understand the content*”), reading purpose (i.e., “*I read to deepen knowledge on my areas of interest*”), and reading environment (i.e., “*I read a lot of books from the library*”). A total of 14 statements were selected from the original scale and measured on a 5-point Likert scale (1: strongly disagree, 5: strongly agree). A higher score reflects a higher level of the child’s reading motivation. Cronbach’s α coefficients of the subfactors are as follows: reading attitude (0.80), reading purpose (0.58), reading environment (0.62).

#### 3.3.4. Academic Achievement

Children’s academic achievement scores were collected by parental self-reports. We used Jung, Kim, Eun, and Choi’s [42] scale to measure children’s grades in five academic subjects (language arts, math, social studies, science, and math) with a 5-point Likert scale (1: very low, 5: very high). A higher score represents higher academic achievement levels.

### 3.4. Statistical Analysis

SPSS 23.0 and AMOS were used for data analysis. Frequency analysis was performed to measure the sociodemographic characteristics of subjects. Next, correlation analysis was conducted to observe the association between key variables (parent–child interaction using newspaper, reading motivation, and academic achievement). A structural equation model was examined afterwards to identify the structural relationship between key variables (parent–child interactions, children’s reading motivation, and academic achievement). As the final step, bootstrapping analysis was conducted to test the significance of the mediating effect. The number of bootstrap samples was 5000 and the percentile confidence level was 95%.

## 4. Results

### 4.1. Newspaper Subscription and Reading

The status of newspaper subscription and reading rates among 1361 households are illustrated below in Table 2. 88.17% of children did not read children’s printed newspapers and less than 10% of them subscribed to children’s newspapers. In addition, 10.43% of children read online newspapers, which was slightly lower than 12.83% who read children’s paper newspapers. Thus, a majority of elementary school students do not read newspapers with low subscription rates. As for the parents, 25.42% of them read printed newspapers and 15.50% subscribed to printed newspaper. 86.70% of parents read online newspapers. These figures show that although the subscription rate is somewhat higher than that of children’s newspapers, only a low percentage of families are subscribed to adult newspapers. The high rate of online reading indicates that reading on on digital devices has become a common practice alternative to paper reading.

### 4.2. Correlation between Parent–Child Interactions Using Newspapers, Reading Motivation, and Academic Achievement

The correlations between variables and descriptive statistics are shown in Table 3 below. A total of 238 households with children in third to sixth grade were observed. For detailed analysis, parent–child interaction and reading motivation were analyzed at subfactor levels. All values fell within the acceptable range for normal distribution, with absolute values of skewness lower than 2 and absolute values of kurtosis higher than 7. The correlations between the variables show the following (Table 3).

Most of the subfactors of parent–child interactions using newspapers showed positive correlation with children’s reading motivation. Parent–child interaction had a positive correlation with most subfactors of academic achievement, including language arts, social studies, science, and English, whereas no correlations were found with math. Reading motivation demonstrated a positive correlation with all subjects of academic achievement.

### 4.3. Structural Relationship between Parent–Child Interactions Using Newspapers, Reading Motivation, and Academic Achievement

#### 4.3.1. Model Fit Evaluation

Before running a path analysis of the model, confirmatory factor analysis was conducted to verify the model’s goodness of fit [43]. The model demonstrated acceptable fit, CFI = 0.95, TLI = 0.93, NFI = 0.93, SRMR = 0.08.

#### 4.3.2. Construct Validity Evaluation of Parent–Child Interactions Using Newspapers, Reading Motivation, and Academic Achievement

Table 4 below shows the convergent validity and discriminant validity of parent–child interactions using newspapers, reading motivation, and academic achievement. First, standardized coefficients of regression weights of these three latent variables were found significant ranging between 0.63 and 1.00 (*p* < 0.001), AVE value higher than 5, construct validity over 0.7 all confirmed the construct validity of the scales used. Next, comparison of the AVE value and correlations of latent variables show that AVE value was higher than the correlation values indicating good discriminant validity.

#### 4.3.3. Structural Equation Modeling

**Figure 1:**
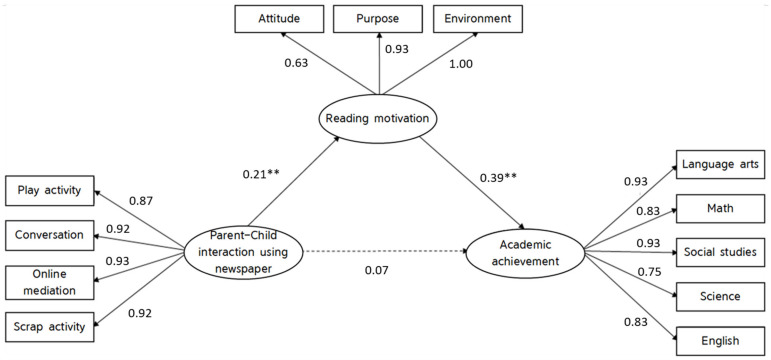
Standardized estimates of structural equation model. Note. The solid line refers to a significant route, and the dotted line is insignificant. All routes are standardized coefficients. ** *p* < 0.01.

As confirmed above, model shows an appropriate fit (CFI, TLI = 0.93, NFI = 0.93, SRMR = 0.08). Figure 1 below illustrates the results of path analysis of structural associations between parent–child interactions using newspapers, children’s reading motivation, and academic achievement.

The R^2^ value, which represents the explanatory power of variables related to academic achievement shows that that parent–child interaction using newspapers explains 4.5% of reading motivation and that parent–child interaction using newspapers explains 16.8% of academic achievement. Next, the direct effects of the model showed that parent–child interaction using newspapers had a significant effect on child’s reading motivation (*β* = 0.21, *p* < 0.01), and child’s reading motivation had a significant effect on academic achievement (*β* = 0.39, *p* < 0.001).

Finally, bootstrapping analysis was performed to test the significance of the indirect effect (refer to Table 5 below) [43]. The mediating effect of reading motivation in the association between parent–child interaction and academic achievement was found to be significant (*β* = 0.08, *p* < 0.01). Since the direct effect of parent–child interaction was insignificant, it can be said that reading motivation is fully mediating the relationship between parent–child interaction and academic achievement. Thus, parent–child interaction using newspapers indirectly affects child’s academic achievement through mediating role of child’s reading motivation.

## 5. Discussion

The present study explored recent status of newspaper subscriptions and reading rates in families with elementary students and examined the structural relationship between parent–child interactiong using newspapers, children’s reading motivation, and academic performance. Below are the main findings of the study.

### 5.1. Status of Newspaper Subscriptions and Reading at Home with School-Age Children

Regarding the status of newspaper subscriptions and reading at home with school-age children, we found that most parents read online newspapers, with less than 30% of parents reading paper newspapers with a 17% subscription rate. For elementary school children, 1 in 10 are subscribed to children’s paper newspapers and 12.9% and 11.5% of children read paper or online newspapers, respectively. Contrary to their parents, there was a low rate of online reading in children, indicating that online newspapers are mainly read by adults. The low number of children reading online newspapers may be due to less available newspaper content for the target audience. Although the benefits of leisure reading have been noted in literature [19], there is a relatively a low number of children who do not read in general. Infrequent readers should receive more parental encouragement to read newspapers as increasing reading amount can be achieved through physical access and discussion opportunities [44]. As for parents and children who do not read newspapers, understanding the ways in which they perceive newspapers would allow future educators to be more considerate to the needs of families in encouraging their participation with newspapers as part of home literacy activities.

The reading and subscription trends found in this study are consistent with the media type survey conducted in 2017 [17] showing an increase in online newspaper readership and a decrease in paper newspaper subscription. With digital technology dominating the news market, online newspaper reading has become the most common news consumption outlet. These trends show that parents with school-aged children may have less access to paper newspapers, and therefore, lack familiarity with using paper newspapers for pedagogical purposes. As our results show that newspapers can function as literacy tools in homes, education on effectively utilizing newspapers is needed for both parents and children.

Furthermore, in contrast to the common notion that “digital natives” prefer electronic over paper newspapers [45], our study showed that slightly more children read paper newspapers than online newspapers. This may be because our participants included first and second graders, who are less likely to access digital devices than older children. The results also suggest the need to look into online newspaper content for young school-aged children.

### 5.2. Correlation between Parent–Child Interactions Using Newspapers, Children’s Reading Motivations, and Academic Achievements

The correlations between parent–child interactions using newspapers, children’s reading motivation, and academic achievement revealed the following. Of the five academic achievement variables, all subfactors of parent–child interactions were positively correlated with language arts, while only conversational and online mediation was positively correlated with social studies. This is in parallel to earlier research that revealed positive effects of newspapers in developing linguistic competence including reading, speaking, listening, and writing skills, along with cultivating morality [2,4,6,46]. Hart and Risley (1999) note that successful linguistic development occurs based on adult–child conversations in a natural form of a “social dance” [47]. In this sense, discussing newspaper content can function as a scaffolding device for children’s language development, as engaging in verbal interactions with adults creates opportunities for children to explore their thoughts.

There was no correlation between parent–child interaction and math, while science and English were only positively correlated with conversational mediation. Such finding is contrary to prior research that shows positive implications of newspapers in teaching mathematics, science, and English [5,7,8,9,13]. While newspaper contents raises issues and deliver real-world information, they do directly pass on knowledge. It can be difficult for school-aged children to make explicit connections just by reading newspaper articles, particularly for academic subjects that require deeper understanding of technical concepts. Thus, parents can guide children in making these connections by engaging in specific interactional activities relevant to the target information. For example, parents may encourage children to play with numbers or explore information related to scientific experiments in news articles.

Regarding reading motivation, most subfactors of the parent–child interaction variable were positively correlated with the subfactors of reading motivation. This finding is consistent with various studies that show the importance of parental roles at home in children’s literacy development [1,20,22,27,34,35]. This is also in line with socio-cultural theories that support the crucial role of parents in children’s learning and cognitive development. Parental engagement in play-based activities and verbal discussions with their school-aged children can improve children’s reading motivation. Of the subfactors of parent–child interaction variable, online mediation showed the strongest correlation while scrap activity, which requires physical contact with paper newspapers, showed the weakest correlation. Results show the continued significance of online mediation activities for children. Indeed, digital literacy education is essential for young children, as they are vulnerable to unfiltered information. A study on American adolescents showed that the majority of adolescents could not differentiate between false and authentic news [48]. This is concerning given that young children are more likely to experience difficulties in critically evaluating online resources on their own. Digital literacy should be fostered through learning how to search and select appropriate online newspaper articles.

As for the correlations between children’s reading motivation and academic achievement, all subfactors of reading motivation were positively correlated with the five school subjects (language arts, math, social studies, science, and math). This is parallel to prior studies that show the close relationship between learner’s reading motivation and academic performance [38,49]. It is presumed that highly motivated children do not avoid challenges and persist toward their goals during the learning process [50], increasing the likelihood of obtaining the target knowledge. Taken together, the findings for our second research question support the theoretical validity of our research model (reading motivation as a possible mediating factor between parent–child interactions and academic achievement).

### 5.3. Structural Relationship between Parent–Child Interactions Using Newspapers, Children’s Reading Motivations, and Academic Achievements

The analysis of the structural model shows that children’s reading motivation fully mediates the relationship between parent–child interactions and children’s academic performance. In other words, parental interactions do not directly affect children’s academic performance, but rather through an indirect pathway, by increasing children’s reading motivation. For elementary school students, promoting reading motivation by familiarizing them with reading through newspapers is an effective way to improve their academic performance rather than employing newspapers as the sole source of knowledge. As children advance in their grade level, they need diverse literacy experiences outside of school contexts in addition to reading textbooks. At homes, newspapers can be utilized as “living textbooks” [51], engaging children with real-world issues and promoting learning interests. As children’s reading motivation has also been found to decrease with age [52,53,54], it is important to utilize literacy sources to maintain their motivation.

The theory of intrinsic and extrinsic motivation suggests that intrinsically motivated children make efforts to attain certain goals and make use of various cognitive strategies [52,55]. From this perspective, children with high reading motivation would activate more cognitive strategies, engage in more efficient learning, and thereby, attain higher academic achievement. Initiated interest through newspaper reading can expand to children’s overall reading experience. Hence, caregivers should actively engage in interactional activities and encourage newspaper reading with young children, especially because childhood reading habits are maintained throughout adulthood [56].

Taken together, newspapers serve as a valuable learning tool, even outside of classroom contexts. We do note that the regression coefficient value from parent–child interactions using newspapers as reading motivation is moderately low (0.21); however, we presume that this is because home literacy environment and parent–child interaction are more general predictors of reading motivation [34,35,36], whereas parent–child interactions using newspapers is a very specific predictor that explains reading motivation as part of the HLE. Parents should place value on literacy activities providing access to newspapers and interactional opportunities. NIE programs in schools should also be utilized in parallel to newspaper reading in homes to maximize educational benefits. In this regard, parental educational programs can help foster children’s newspaper reading behaviors. In a meta-analysis of 20 intervention programs for third graders, parents’ participation had a positive impact on children’s literacy development [57]. One study in this meta-analysis showed that parental interventions to help guide their children’s reading methods were twice as effective in developing children’s literacy. These intervention programs were found to be equally effective for children at risk of reading disabilities, regardless of their socioeconomic factors, suggesting the need for NIE programs. To adequately support children’s literacy development, family literacy education is the most ideal learning intervention for developing children [57]. Therefore, family-level efforts need to be made to ensure a positive learning process.

## 6. Conclusions

The current study explored the status of newspaper subscriptions and reading in families with school-aged children. Furthermore, this work analyzed information on online newspaper reading to reflect upon the current trends in the media landscape. Additionally, by examining the structural relationship between parent–child interactions, children’s reading motivation, and academic achievement, this work supports the significance of newspapers, particularly the importance of parental online mediation. Findings suggest practical ways to allow parents to better understand how to implement newspapers in their teaching practices by reviewing concrete types of parent–child interactions by using newspapers.

There are a few noteworthy limitations to this study. First, children’s academic achievements were measured through parental self-reports, as the study was conducted online in a national-scale survey. Future studies should measure academic performance in a more objective manner, such as by measuring student’s test scores. Second, convenience sampling was used as the sampling technique. While convenience sampling has its advantages, this method has less generalizable samples than probability samples. Parents of children who have a greater interest in utilizing newspapers and children’s education may have been over-collected, and thus, caution is required when interpreting the findings.

## Figures and Tables

**Table 1:** Sociodemographic characteristics of subjects (*N* = 1361).

Subject	Division	Classification	Number (*n*)	Percentage (%)
Child	Gender	Male	694	51.99
		Female	667	49.01
	Grade	1st	360	26.45
		2nd	280	20.57
		3rd	187	13.74
		4th	198	14.55
		5th	198	14.55
		6th	138	10.14
Parent	Father’s level of education	High school graduate	87	6.39
		2–3 year college graduate	102	7.49
		Undergraduate	702	51.58
		Graduate	470	34.53
	Mother’s level of education	High school graduate	83	6.10
		2–3 year college graduate	135	9.92
		Undergraduate	748	54.96
		Graduate	348	25.57
	Job status	Dual earner	1013	74.43
		Sole earner (Father)	329	24.17
		Sole earner (Mother)	15	1.10
		Unemployed	4	0.29
	Average monthly household income	Less than KRW 2 million	10	0.73
		KRW 2–3 million	125	9.18
		KRW 4–5 million	360	26.45
		KRW 6–7 million	476	35.97
		KRW 8–10 million	217	15.94
		Over KRW 10 million	173	12.71

**Table 2:** Newspaper subscription and reading (*N* = 1361).

Subject	Category		Classification	Frequency (*n*)	Percentage (%)
Child	Printed newspaper for children	Subscription	Yes	124	9.11
			No	1237	90.89
		Reading	Yes	161	12.83
			No	1200	88.17
	Reading online newspaper for children		Yes	142	10.43
			No	1219	89.57
Parent	Printed newspaper	Subscription	Yes	211	15.50
			No	1150	84.50
		Reading	Yes	346	25.42
			No	1015	74.58
	Reading online newspaper		Yes	1180	86.70
			No	181	13.30

**Table 3:** Frequency and correlation relationship of variables (*N* = 238).

Variables		1	2	3	4	5	6	7	8	9	10	11	12
Reading motivation	1 Attitude	1											
	2 Purpose	0.58 ***	1										
	3 Environment	0.63 ***	0.93 ***	1									
Academic achievement	4 Language arts	0.54 ***	0.37 ***	0.40 ***	1								
	5 Math	0.49 ***	0.34 ***	0.37 ***	0.75 ***	1							
	6 Social studies	0.48 ***	0.36 ***	0.40 ***	0.76 ***	0.63 ***	1						
	7 Science	0.45 ***	0.30 ***	0.32 ***	0.73 ***	0.68 ***	0.89 ***	1					
	8 English	0.42 ***	0.31 ***	0.32 ***	0.73 ***	0.71 ***	0.75 ***	0.76 ***	1				
Interaction using newspapers	9 Play activity	0.17 **	0.16 *	0.20 **	0.13 *	−0.01	0.09	0.04	0.02	1			
	10 Conversation	0.20 **	0.17 **	0.19 **	0.21 **	0.05	0.21 **	0.17 *	0.13 *	0.77 ***	1		
	11 Online mediation	0.21 **	0.22 **	0.25 **	0.20 **	0.05	0.19 **	0.13	0.11	0.83 ***	0.86 ***	1	
	12 Scrap activity	0.13 *	0.11	0.14 *	0.14 *	−0.01	0.11	0.06	0.07	0.81 ***	0.86 ***	0.84 ***	1
M		3.75	3.45	3.40	5.23	5.22	5.19	5.24	5.20	1.46	1.81	1.57	1.61
SD		0.59	0.91	0.86	0.80	0.83	0.84	0.81	0.88	0.88	1.21	0.97	1.06
Skewness		−0.87	−0.28	−0.30	−1.00	−1.11	−0.90	−1.09	−1.09	2.00	1.16	1.59	1.62
Kurtosis		0.92	−0.38	−0.05	1.29	1.39	0.50	0.93	1.13	3.40	−0.06	1.45	1.48

* *p* < 0.05; ** *p* < 0.01; *** *p* < 0.001.

**Table 4:** Construct validity.

			B	*β*	C.R.	AVE	Construct Reliability	φ^2^
A. Parent–child interaction using newspapers	→	Play	0.78	0.87	20.78 ***	0.84	0.95	A ↔ B = 0.04 A ↔ C = 0.00 B ↔ C = 0.24
		Verbal intervention	1.14	0.92	24.52 ***			
		Online intervention	0.92	0.93	25.17 ***			
		Scraping newspaper	1.00	0.92				
B. Reading motivation	→	Attitude	1.00	0.63		0.64	0.83	
		Purpose	2.30	0.93	12.02 ***			
		Environment	2.32	1.00	12.59 ***			
C. Academic achievement	→	Language arts	1.00	0.83		0.61	0.88	
		Math	0.85	0.75	13.49 ***			
		Social studies	1.32	0.93	18.67 ***			
		Science	1.29	0.93	18.72 ***			
		English	1.19	0.83	15.59 ***			
Standard				>0.6		>0.5	>0.7	AVE > φ^2^

Note. C.R. = Critical Ratio, AVE = Average Variance Extracted, φ^2^ = Squared correlation coefficients between latent variables, *** *p* < 0.001.

**Table 5:** Bootstrapping analysis.

Pathway	Total	Direct	Indirect
parent–child interaction → reading motivation → academic achievement	0.15 *	0.07	0.08 **

* *p* < 0.05, ** *p* < 0.01.

## Data Availability

The datasets generated during during the present study are available from the corresponding author upon reasonable request.
